# Reassembly of Nucleosomes at the *MLH1* Promoter Initiates Resilencing Following Decitabine Exposure

**DOI:** 10.1371/journal.pgen.1003636

**Published:** 2013-07-25

**Authors:** Luke B. Hesson, Vibha Patil, Mathew A. Sloane, Andrea C. Nunez, Jia Liu, John E. Pimanda, Robyn L. Ward

**Affiliations:** Adult Cancer Program, Lowy Cancer Research Centre and Prince of Wales Clinical School, University of New South Wales, Sydney, New South Wales, Australia; University of Washington, United States of America

## Abstract

Hypomethylating agents reactivate tumor suppressor genes that are epigenetically silenced in cancer. Inevitably these genes are resilenced, leading to drug resistance. Using the *MLH1* tumor suppressor gene as a model, we showed that decitabine-induced re-expression was dependent upon demethylation and eviction of promoter nucleosomes. Following decitabine withdrawal, *MLH1* was rapidly resilenced despite persistent promoter demethylation. Single molecule analysis at multiple time points showed that gene resilencing was initiated by nucleosome reassembly on demethylated DNA and only then was followed by remethylation and stable silencing. Taken together, these data establish the importance of nucleosome positioning in mediating resilencing of drug-induced gene reactivation and suggest a role for therapeutic targeting of nucleosome assembly as a mechanism to overcome drug resistance.

## Author Summary

Hypomethylating agents are emerging as effective cancer therapies. However, their therapeutic effects are transient and drug resistance inevitably develops. While resistance is associated with resilencing of genes initially demethylated by the drug, the mechanism underlying this resilencing is unknown. We provide evidence that the rapid reassembly of nucleosomes at transcription start sites initiates resilencing and is a prerequisite for promoter remethylation. This finding shows reassembly of nucleosomes at the promoter of critical genes is a potential early marker of resistance to hypomethylating agents. Our findings have implications for the treatment of cancer using epigenetic therapies that target DNA methylation alone, and suggest that overcoming drug resistance will require therapeutic strategies which prevent nucleosome deposition.

## Introduction

The DNA hypomethylating agents decitabine (5-aza-2′deoxycytidine) and azacitidine (5-azacytidine) are established therapies for myeloid malignancies and show promise in treating solid tumors [1]. These drugs are cytidine analogs that covalently trap the DNA methyltransferase I (DNMT1 [NP_001124295]) protein onto DNA, targeting the enzyme for proteasome degradation. The resulting depletion of DNMT1 leads to passive demethylation in dividing cells. The observed effects of low dose decitabine on cell growth, differentiation [2] and enhanced immunological responses to tumor-associated antigens [3] are thought to be due to the re-expression of critical genes silenced by aberrant promoter hypermethylation. Sustained gene re-expression has been associated with clinical response [4], [5], supporting the view that it is critical to the therapeutic mechanism of action of these drugs. Clinically relevant low doses of decitabine and azacitidine can lead to sustained changes in gene expression that are associated with reduced tumorigenicity in mice bearing transplanted tumor xenografts [6].

Like all anti-cancer therapies, resistance to hypomethylating agents ultimately develops, and without alternative therapies patients succumb quickly to their disease [7]. A variety of mechanisms have been proposed to explain resistance, including insufficient drug uptake by membrane transporters, deficiency of the enzyme required for drug activation (deoxycytidine kinase), or increased drug metabolism through deamination by cytidine deaminase [8]. Although these mechanisms may explain the resistance of some cell lines *in vitro* [8], a recent study showed they do not explain acquired resistance in patients [9].

Numerous *in vitro* studies show that gene re-expression following decitabine treatment is transient [10], [11]. The silencing of genes initially re-expressed by decitabine treatment is termed gene resilencing [12], [13]. If sustained gene re-expression correlates with clinical response then gene resilencing is likely to play a role in the development of drug resistance. Therefore, understanding the mechanistic basis of gene resilencing is a prerequisite for the development of therapies that cause sustained gene re-expression and prolonged clinical response. The rapid onset of gene resilencing is unlikely to be explained by DNA remethylation, which *in vitro* studies have shown occurs gradually over several weeks [11], [13]. Also drug resistance occurs despite persistence of DNA hypomethylation [9]. This suggests other epigenetic mechanisms, such as nucleosome positioning or histone modifications, are responsible for driving the resilencing of genes.

We reasoned that promoter nucleosome positioning could explain gene resilencing independently of DNA remethylation. To address this hypothesis we mapped the temporal onset of epigenetic changes at the *MLH1* [NM_000249] gene promoter following exposure of RKO cells to decitabine. We used this model because the *MLH1* gene is biallelically methylated and silent in this cell line and activation of the promoter with decitabine has been shown to involve nucleosome eviction [14].

## Results

### Promoter remethylation is a late event in the resilencing of *MLH1*

**Figure 1:**
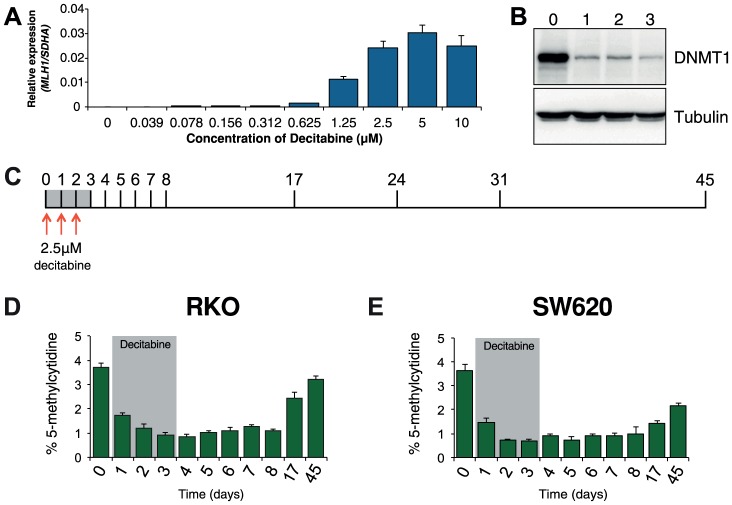
Optimization of decitabine treatment. **A**, qRTPCR results showing *MLH1* gene expression in RKO cells after treatment with the indicated concentration of decitabine. Cells were treated every 24 hours for a period of 72 hours. *MLH1* expression was normalized to *SDHA*. **B**, Immunoblot of DNMT1 protein in total protein lysates from RKO cells treated with 2.5 µM decitabine for the indicated number of days. **C**, Optimized decitabine dosing schedule, treatment period (gray box) and days on which cells were harvested. **D** and **E**, Global methylation analysis using LC-MS/MS in RKO and SW620 cells treated with decitabine. Error bars = SD.

Optimization experiments showed that 72 hours of decitabine treatment at a concentration of 2.5 µM led to near maximal re-expression of *MLH1* and depletion of DNMT1 protein in RKO cells (Figure 1A, B). Using these conditions we profiled global methylation changes at baseline (day 0), as well as throughout decitabine treatment (days 1–3) and during recovery (days 4–45, Figure 1C). As expected, we observed a decrease in global methylation from 3.7%±0.2 in untreated RKO cells to 0.8%±0.1 at day 4. Global methylation levels remained low up to day 8 and recovered gradually to near baseline levels of 3.2%±0.2 by day 45 (Figure 1D). In SW620 cells, global remethylation was also observed although it occurred more slowly than in RKO cells (Figure 1E).

**Figure 2:**
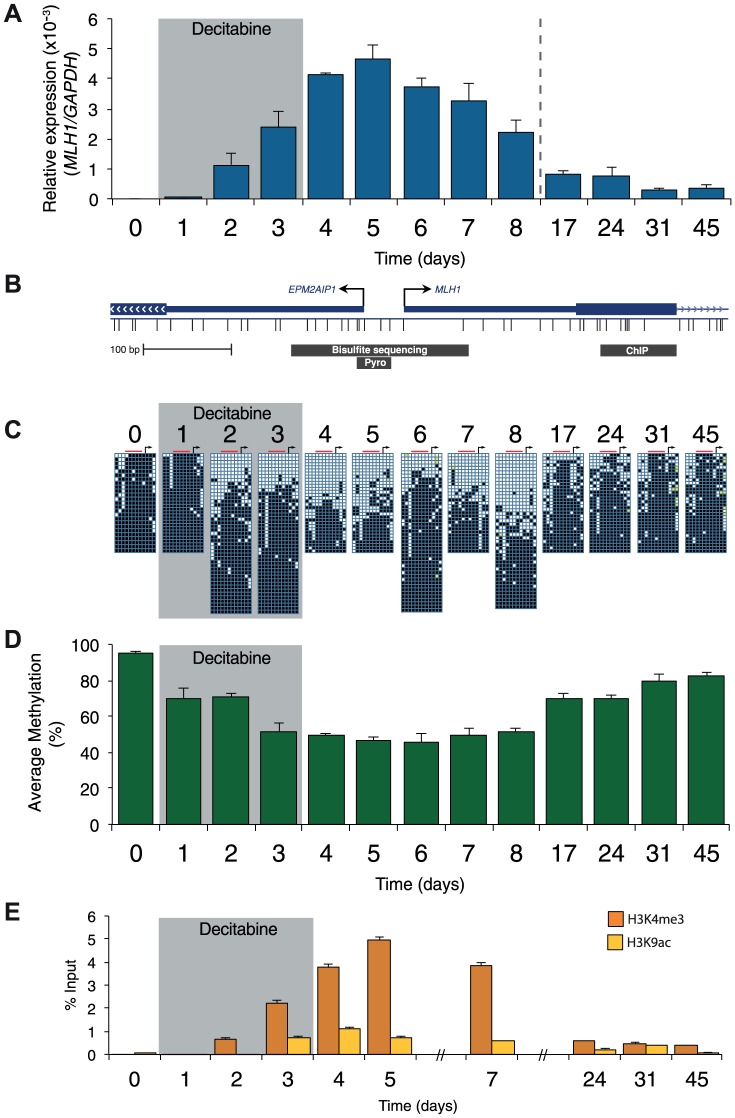
Resilencing precedes promoter remethylation. **A**, qRTPCR results showing *MLH1* gene expression in RKO cells normalized to *GAPDH*. **B**, Schematic of the *MLH1* promoter indicating the regions assayed by allelic bisulfite sequencing, bisulfite pyrosequencing (Pyro) and ChIP for 2C, D and E, respectively. **C**, Allelic bisulfite sequencing across the *MLH1* promoter. Black squares = methylated CpG dinucleotides, white squares = unmethylated CpG dinucleotides, yellow filled squares = not determined. Black arrow = *MLH1* TSS. Red bar indicates the location of sites assayed using bisulfite pyrosequencing. **D**, Bisulfite pyrosequencing showing average percentage methylation levels across 5 CpG sites upstream of the *MLH1* annotated TSS. **E**, ChIP qPCR results showing enrichment of H3K4me3 and H3K9ac. Error bars = SD. See also Figure S1.

Prior to treatment, *MLH1* mRNA levels in RKO cells were undetectable (Figure 2A). Methylation analysis of the regions indicated in Figure 2B showed 95% methylation by pyrosequencing, whilst allelic bisulfite sequencing showed hypermethylation on 100% (25/25) of promoter molecules (Figure 2A, C and D). Two days after withdrawal of decitabine (day 5) *MLH1* mRNA reached maximal levels (Figure 2A). This coincided with a near maximal decrease in promoter methylation, which dropped to 46.3% at day 5 (Figure 1D), with 31.3% (10/32) molecules showing complete or near complete demethylation (defined as no more than two methylated CpG dinucleotides per molecule; Figure 2C). Having demonstrated that short-term exposure to decitabine had re-expressed *MLH1* we then determined the temporal onset of *MLH1* resilencing in RKO cells by profiling *MLH1* mRNA levels up to day 45. The initial stages of resilencing (defined as the point when mRNA levels begin to decrease after decitabine withdrawal) started at day 6, and by day 17 expression levels were only 17.5% of the level of maximal *MLH1* expression observed at day 5 (Figure 2A). However, throughout this period (up to day 8) methylation levels remained low. For example, by day 8 methylation levels were very similar to day 5 at 51.3%, with 35.6% (16/45) demethylated promoter molecules despite mRNA levels declining by over 50% (Figure 2A, C, D). These results show that promoter remethylation does not precede or coincide with *MLH1* resilencing. Interestingly, when remethylation did occur (between days 8 and 17), we found preferential methylation of five CpG sites immediately upstream of the *MLH1* transcription start site (TSS; Figure 2C) that are critical to the regulation of expression [15]. In SW620 cells we also observed fluctuations in *MLH1* expression levels (between 1.5-fold and 0.4-fold relative to baseline at day 0) during and after decitabine exposure, though these changes were not related to DNA methylation (Figure S1).

We next profiled histone modification changes using native ChIP, focusing on the ‘active’ histone marks H3K9ac and H3K4me3 (Figure 2E and Figure S1C). For control experiments we measured the levels of these histone modifications at the *GAPDH* [NM_002046] promoter (Figure S1C). Prior to treatment with decitabine the levels of H3K9ac and H3K4me3 at the *MLH1* promoter were either very low or undetectable (Figure 2E). Levels of H3K9ac and H3K4me3 increased and decreased with a very similar trend to the re-expression and resilencing of *MLH1*. By contrast, in SW620 cells the levels of H3K4me3 and H3K9ac at the *MLH1* promoter were comparable with levels at the *GAPDH* promoter as expected due to the high levels of *MLH1* expression in this cell line (Figure S1C). We also found the levels of the ‘repressive’ histone modification H3K27me3 remained very low or undetectable at the *MLH1* promoter throughout treatment in RKO cells (data not shown). As a control for H3K27me3, we used primers specific for the *MYOD1* [NM_002478] promoter, which is enriched for this histone modification.

Taken together these data confirm that *MLH1* re-expression is accompanied by promoter DNA demethylation and acquisition of the active histone marks H3K9ac and H3K4me3. These data also demonstrate that *MLH1* re-expression is tightly linked to DNA demethylation whereas the early stages of resilencing are independent of DNA remethylation.

### Nucleosome levels at the *MLH1* promoter rapidly recover following decitabine withdrawal

**Figure 3:**
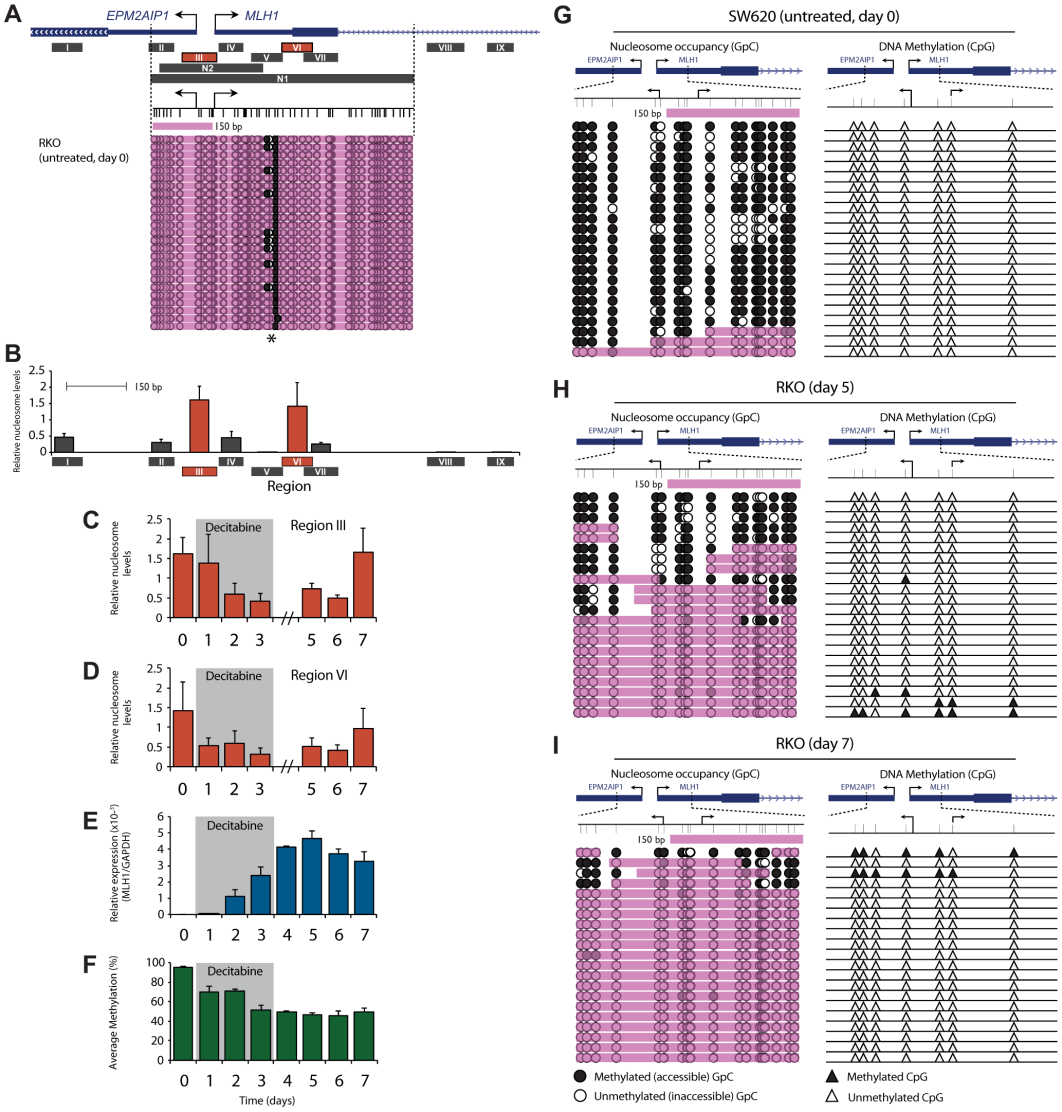
Nucleosome reassembly at the TSS is the initiating event in *MLH1* resilencing. **A**, Regions assayed for nucleosome occupancy using MNase-qPCR (Regions I–IX) and NOMe-Seq (Regions N1 and N2). Shown beneath the gene schematic is NOMe-Seq data from untreated RKO cells at Region N1. Black arrows indicate the *MLH1* and *EPM2AIP1* TSS. Bottom panel represents GpC accessibility. Black circles = GpC dinucleotides methylated/accessible to the GpC methyltransferase M.*Cvi*PI. White circles = GpC dinucleotides not methylated/inaccessible to GpC methyltransferase. Pink shading indicates regions of inaccessibility of ≥150 bp. Asterix = region of M.*Cvi*PI accessibility. **B**, Relative nucleosome levels in untreated RKO cells at the indicated regions (black bars labeled Regions I–IX) as determined by MNase-qPCR. Drawn to scale with schematic shown in A. Error bars = SD. **C** and **D**, qPCR results showing changes in relative nucleosome levels at Regions III and VI following decitabine exposure. **E** and **F**, *MLH1* gene expression (E) and promoter bisulfite pyrosequencing (F), reproduced from Figure 2A and D for ease of comparison with nucleosome levels. **G–I**, NOMe-Seq analysis of the *MLH1* promoter at Region N2 in SW620 (F) and RKO (G,H) cells at the indicated treatment points. Black filled triangles = methylated CpG dinucleotides; white filled triangles = unmethylated CpG dinucleotides. See also Figure S2.

Having demonstrated the relationship between *MLH1* expression, promoter demethylation and active histone modifications, we sought to determine how nucleosome levels across the promoter change during and after decitabine exposure. We firstly determined nucleosome levels and positioning in untreated RKO cells. This was done using MNase digestion coupled with qPCR (MNase-qPCR) at nine regions across the *MLH1* promoter (Regions I–IX; Figure 3A), as well as with Nucleosome Occupancy and Methylome Sequencing (NOMe-Seq; Region N1 in Figure 3A). This showed dense nucleosome occupancy across the *MLH1* promoter and precisely mapped the positions of two nucleosomes, one at the *MLH1* TSS and one within exon 1 (Figure 3A, B). This was evident from a small region of DNA accessible to the GpC methyltransferase M.*Cvi*PI (asterix, Figure 3A) and MNase (Region V, Figure 3B) indicating a region of linker DNA between two adjacent and precisely positioned nucleosomes. Furthermore, MNase-qPCR detected strong nucleosome positioning at Regions III and VI flanking this site of MNase and M.*Cvi*PI accessibility (Figure 3B). Decitabine-induced *MLH1* re-expression was associated with the eviction of these nucleosomes (Figure 3C–E), as well as nucleosomes from all regions across the *MLH1* promoter (Figure S2A–G). By day 3 (final day of decitabine exposure) nucleosome levels at Regions III and VI were lowest at 25.4% and 22.6% respectively, relative to levels in untreated cells. These results confirm that in addition to DNA demethylation, *MLH1* re-expression is dependent upon eviction of promoter nucleosomes, as described in previous reports [14]. In SW620 cells (normally expressing *MLH1*) we found much lower levels of nucleosome occupancy at Regions III and VI. Interestingly, decitabine treatment of SW620 cells also resulted in nucleosome eviction from these regions (Figure S2H), which may explain the initial increase in *MLH1* expression seen in SW620 cells following decitabine exposure (Figure S1A).

Next, we determined how nucleosome levels across the *MLH1* promoter change during the initial stages of resilencing and compared this with the *MLH1* expression and promoter methylation data described above. At day 5, when *MLH1* re-expression was maximal, nucleosome levels across the promoter remained low (Figure 3C–E). Strikingly, by day 7 (just 4 days after decitabine withdrawal) we found that nucleosomes had reoccupied the *MLH1* TSS and exon 1 and that this coincided with the decline of gene expression (Figure 3C–E and Figure S2). Restoration of nucleosome occupancy occurred despite the *MLH1* promoter remaining hypomethylated (Figure 3F) suggesting that the reassembly of nucleosomes at the *MLH1* TSS might initiate gene resilencing, and that this precedes DNA remethylation.

### Nucleosomes reoccupy the demethylated *MLH1* promoter in the initial stages of resilencing

Given our observation that nucleosome deposition occurred in the absence of remethylation it was important to determine nucleosome occupancy and DNA methylation on individual molecules. In doing so, we sought to determine whether nucleosome reassembly occurs on demethylated promoter molecules which would indicate that this was an initiating event in gene resilencing and a prerequisite for remethylation. We designed a second NOMe-Seq assay across the *MLH1* TSS Region N2, Figure 3A). This assay was designed to preferentially amplify unmethylated DNA, allowing us to determine nucleosome occupancy on DNA molecules that had been demethylated by decitabine treatment. Using this assay, we profiled nucleosome occupancy in untreated SW620 cells. We found that 91% (21/23) of molecules were nucleosome depleted across the TSS (Figure 3G) and accordingly, *MLH1* was highly expressed (Figure S1A). Next we analyzed decitabine treated RKO cells at day 5 when *MLH1* re-expression was maximal. This revealed nucleosome eviction from the TSS on a proportion (9/22) of demethylated promoter molecules (Figure 3H) confirming that re-expression was associated with the eviction of nucleosomes, but also suggesting that some demethylated promoter molecules remain nucleosome bound. However, by day 7, when resilencing of gene expression had begun (Figure 3E), NOMe-Seq showed that all demethylated molecules assayed were nucleosome occupied across the TSS (Figure 3I). This clearly shows that nucleosomes reassemble onto demethylated promoter molecules and that nucleosome occupancy rather than DNA methylation is associated with reduced gene expression.

## Discussion

This study shows that *MLH1* resilencing is initiated by a rapid restructuring of chromatin architecture, characterized by the reassembly of nucleosomes at the TSS. This restructuring occurs prior to DNA remethylation, suggesting that gene resilencing following exposure to decitabine is controlled by a hierarchy of epigenetic events.

Whilst previous studies have described decitabine-induced gene reactivation in detail, in this study we specifically focused on the molecular events associated with gene resilencing. This is technically challenging as it requires a model system to determine the temporal relationship between gene expression and promoter epigenetic changes, ideally at daily intervals. It also requires sampling of large numbers of cells to measure each variable at each time point. This renders such experiments impossible using material from patients receiving decitabine. Furthermore, variability in the molecular drivers between patients would make the identification of a model gene a major obstacle. To overcome these difficulties, we chose to track the resilencing of the *MLH1* gene in RKO colorectal carcinoma cells following decitabine exposure. *MLH1* is an archetypal gene inactivated by hypermethylation and loss of expression plays a pivotal role in the development of colorectal and other cancers [16]. This gene has been extensively epigenetically characterized using a variety of *MLH1*-specific assays [14], [17]. Furthermore, RKO cells show biallelic hypermethylation of the *MLH1* promoter allowing us to examine epigenetic changes on a homogeneous population of silent *MLH1* promoter molecules at baseline.

By measuring *MLH1* expression levels at daily intervals we were able to precisely pinpoint the initiation of resilencing. This then allowed us to demonstrate that resilencing began when the *MLH1* promoter remained maximally demethylated, which confirms previous reports that the gradual rate of global and site-specific DNA remethylation cannot explain the swiftness of gene resilencing [11], [13]. Instead, we found that *MLH1* resilencing was tightly linked to nucleosome position and histone modifications. In addition to the loss of H3K4me3 and H3K9ac, we found that nucleosome levels rapidly recovered following decitabine exposure, that nucleosome reassembly coincided with the decline of *MLH1* expression, and that nucleosomes reoccupied the TSS of demethylated molecules. Our data suggest that the reassembly of nucleosomes at the TSS is a prerequisite for remethylation and that this is an important factor in determining the future epigenetic state of the reactivated *MLH1* promoter. A limitation of our study is that these data relate to one cell line and one promoter (*MLH1*), potentially impacting on the generalizability of our findings. However, our proposition that nucleosomes initiate gene resilencing after drug exposure is supported by two recent studies, the first describing differentiation of NCCIT cells and the second describing the silencing of a *GFP* transgene. The study of You *et al.* showed that differentiation of NCCIT cells was associated with nucleosome assembly at the shared *NANOG*/*OCT4* enhancer and that this resulted in the loss of expression of these genes [18]. This study also showed that hypermethylation of the *NANOG* promoter and enhancer followed nucleosome assembly and gene silencing. In the second study, Si *et al.* tracked the resilencing of a *GFP* transgene driven by a cytomegalovirus (CMV) promoter and showed that histone H3 density increased at the CMV promoter five days after withdrawal of decitabine [13].

**Figure 4:**
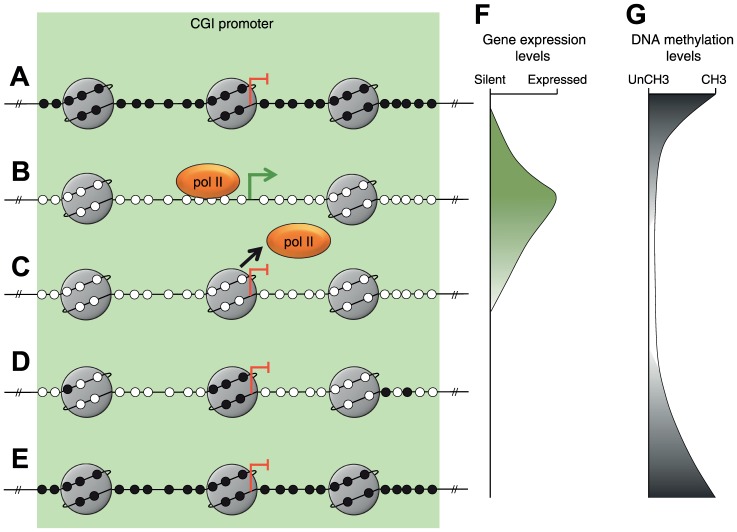
A model of *MLH1* resilencing. **A–E**, Depiction of the sequence of epigenetic changes at the *MLH1* promoter associated with resilencing. Small black filled circles represent methylated CpG dinucleotides. Small white filled circles represent unmethylated CpG dinucleotides. Large gray circles represent nucleosomes. Orange oval represents the RNApolII complex. The green arrow and blunt red arrows indicate the *MLH1* transcription start site and expression status. **F**, **G**, Representation of gene expression and DNA methylation levels associated with each of the stages depicted in A–E, respectively.

By combining our findings with those of previous studies we have constructed a model describing *MLH1* resilencing following decitabine exposure (Figure 4). Prior to treatment, *MLH1* is silent and the promoter is methylated and occupied by nucleosomes (Figure 4A, F and G). Decitabine-induced re-expression is associated with demethylation and nucleosome eviction from the TSS (Figure 4B, F, and G) as shown in this study and by others [14]. Nucleosome eviction from the TSS is associated with increased H3K9ac and H3K4me3 at remaining promoter nucleosomes. We found that trimethylation of H3K4 was tightly linked to *MLH1* expression levels, which agrees with a previous report that H3K4me3 is required for anchoring the TFIID transcription factor subunit of the RNApolII complex [19]. The initial stages of resilencing are associated with the loss of H3K9ac and H3K4me3, which most likely coincides with the loss of RNApolII from the promoter and nucleosome reassembly at the TSS (Figure 4C). Note that gene resilencing occurs when nucleosomes re-enter the promoter on demethylated molecules (Figure 4C, F and G). Finally, gradual remethylation of the *MLH1* promoter over several weeks consolidates the silenced state (Figure 4D, E and G). We found that remethylation occurred preferentially at five CpG sites that overlap with the site of nucleosome reassembly (Figure 4D). The preferential remethylation of these five CpG sites may be explained by a previous report describing the recruitment of DNMT3L-DNMT3A/B complexes to nucleosomes that were unmethylated at lysine 4 of histone H3 [20].

The driving force behind nucleosome reassembly is at present unclear but it may be dependent on the surrounding chromatin context. For example at bivalent promoters, which are characterized by the presence of H3K4me2/3 and H3K27me3, complete and rapid epigenetic repression might be reinstated due to the persistence of H3K27me3 [21]. In our study, we found that H3K27me3 remained very low or undetectable throughout treatment indicating this is unlikely to trigger resilencing at the *MLH1* locus. It is possible however that other histone modifications could be driving gene resilencing prior to remethylation. Interestingly, the persistence of the RNApolII complex at the promoter of *TMS1* is a critical factor in the long-term stability of decitabine-induced reactivation [10]. We propose that at reactivated promoters, equilibrium exists between the binding of the RNApolII complex and the reassembly of nucleosomes. Reduced binding of the RNApolII complex would invite nucleosome reassembly at the TSS, preventing further binding of the RNApolII complex and ultimately leading to promoter remethylation. This hypothesis is consistent with previous reports showing that continued binding of the RNApolII complex protects against *de novo* methylation [22].

A key question is whether gene resilencing is a result of active chromatin re-modeling or clonal expansion of cells that did not respond to decitabine treatment. Although we did not measure cell death in treated cells, we consider two components of our data strongly support our conclusion that resilencing of *MLH1* is an active process. Firstly, nucleosome levels at the *MLH1* promoter recover before DNA methylation levels. This stepwise recovery in chromatin structure argues against passive resilencing, which would be associated with simultaneous re-emergence of repressive chromatin features (methylated and nucleosome occupied DNA). Secondly, single-molecule analysis of the *MLH1* promoter shows that nucleosomes reassemble onto demethylated molecules, and since *MLH1* is biallelically hypermethylated prior to treatment, this shows that we are measuring changes within cells that were demethylated by decitabine exposure.

Our finding that nucleosome occlusion of demethylated promoters can initiate gene resilencing has clear implications for the development of epigenetic therapies. Furthermore, our study raises the possibility that measurement of nucleosome occupancy at the TSS of critical genes may provide a more informative marker of emerging drug resistance than the measurement of promoter methylation.

## Materials and Methods

### Cell culture and decitabine treatment

The colorectal cancer cell lines SW620 (*MLH1* promoter unmethylated and gene expressed) and RKO were maintained in DMEM media supplemented with 25 mM glucose, 10% (v/v) FBS, 100 units penicillin, 100 µg/mL streptomycin and 2 mM glutamate (Life Technologies) and grown at 37°C in 5% CO_2_. Cells were treated every 24 hours for a period of 72 hours by replacing media supplemented with the indicated concentrations of decitabine (5-aza-2′deoxycytidine, Sigma) freshly prepared in 50% filter sterilized acetic acid.

### Gene expression

RNA was extracted using PureLink Micro Kit (Life Technologies). cDNA was prepared using the SuperScript III cDNA Synthesis Kit (Life Technologies) as per the manufacturer's instructions. Real-time quantitative reverse transcriptase PCR (qRTPCR) was performed in triplicates using 10 ng cDNA using iQ SYBR Green supermix (Bio-Rad) and a MyiQ iCycler (Bio-Rad). Please refer to supplementary files Table S1 and Text S1 for primer sequences and sources. Gene expression was normalized to glyceraldehyde-3-phosphate dehydrogenase (*GAPDH* [NM_002046]) or succinate dehydrogenase complex, subunit A (*SDHA* [NM_004168]).

### Immunoblotting

Cells were lysed on ice in 50 mM Tris HCl pH 7.5, 150 mM NaCl, 1% (v/v) Triton X-100, 0.5% (w/v) deoxycholic acid, 0.1% (w/v) sodium dodecyl sulphate (SDS) and EDTA-free Complete Protease Inhibitor (Roche), vortexed and sonicated followed by centrifugation to pellet cell debris. Protein concentration was determined using the bicinchoninic acid protein assay (Pierce) following manufacturer's instructions. Proteins were resolved by SDS-PAGE, transferred to PVDF membrane (Millipore) and probed with 1 µg/mL anti-DNMT1 (R & D systems) or 9.6 ng/mL anti-α-tubulin (Cell Signaling Technology) before incubation with anti-IgG HRP. Proteins were visualized by enhanced chemiluminescence using Image Quant TL software and an Image Quant LAS 400 (GE).

### DNA methylation analysis

#### Allelic bisulfite sequencing

DNA was extracted in 10 mM Tris HCl pH 7.8, 1 mM EDTA, 100 mM NaCl, 1% (w/v) SDS, treated with Proteinase K and purified by phenol chloroform extraction and ethanol precipitation. Sodium bisulfite modification was performed using the EZ DNA methylation Gold Kit (Zymo Research) according to manufacturer's instructions. The *MLH1* CpG island promoter region was amplified from 40 ng of bisulfite treated DNA using the primers listed in Table S1. PCR products were cloned by ligation and transformation using the TOPO TA Cloning kit (Invitrogen). Individual molecules were isolated from transformed colonies by colony PCR before sequencing using BigDye Terminator v3.1 (ABI) and an ABI3730 genetic analyzer (ABI).

#### Bisulfite pyrosequencing

Bisulfite pyrosequencing of 5 CpG sites immediately upstream of the *MLH1* transcription start site was performed as described previously [23]. Each time point was analyzed in quadruplicate.

#### Liquid chromatography tandem mass spectrometry (LC-MS/MS)

Absolute quantities of 5-methyl-2′-deoxycytidine (5mdC; global methylation) were determined using LC-MS/MS as we have described previously [24], [25]. Each time point was analyzed in quadruplicate.

### Native chromatin immunoprecipitation (ChIP)

Native ChIP was performed following micrococcal nuclease digestion of chromatin as described previously [26]. Relative enrichment of histone modifications were assessed using real-time quantitative PCR (qPCR) with primers listed in Table S1. Primers specific to *GAPDH* and *MYOD1* were used as controls for the enrichment of H3K4me3/H3K9ac and H3K27me3, respectively. Enrichment was normalized to undigested input DNA after subtracting non-specific binding determined using pre-immune IgG.

### Isolation of mononucleosome DNA using micrococcal nuclease (MNase) and qPCR

A total of 1×10^7^ cells were lysed on ice in 50 mM Tris HCl pH 7.9, 100 mM KCl, 5 mM MgCl_2_, 50% (v/v) glycerol, 1.5% (v/v) β-mercaptoethanol, 0.1% (w/v) Saponin and Complete Protease Inhibitor with EDTA (Roche) followed by equilibration in 50 mM Tris HCl pH 7.5, 0.32 mM sucrose, 4 mM MgCl_2_, 1 mM CaCl_2_, and EDTA-free Complete Protease Inhibitors (Roche). Chromatin was digested using 20 U MNase (Fermentas) for 4 min at 37°C to achieve maximal digestion to mononucleosomes. Digestion was stopped by the addition of 20 mM EDTA pH 8 and placed immediately on ice. Cellular debris was pelleted and the supernatant treated with Proteinase K before isolation of DNA by phenol chloroform extraction and ethanol precipitation. Mononucleosomal DNA corresponding to 150 bp was isolated by gel extraction using a QIAquick gel extraction kit (Qiagen). DNA concentration was measured using the Quant-iT PicoGreen dsDNA Assay Kit (Invitrogen). Relative nucleosome levels were measured at nine sites at the *MLH1* promoter (designated Regions I–IX) using primers listed in Table S1. Nucleosome levels at each site were normalized to naked genomic DNA.

### Nucleosome occupancy and methylome sequencing (NOMe-Seq)

NOMe-Seq was performed as described previously [27]. This involved treatment of intact nuclei with 200 U GpC methyltransferase M.*Cvi*Pl for 15 min at 37°C followed by termination of the reaction with an equal volume of 20 mM Tris HCl pH 7.9, 600 mM NaCl, 1% (w/v) SDS and 10 mM EDTA, and isolation of DNA as described above. DNA was bisulfite converted and amplified using primers listed in Table S1. M.*Cvi*PI enzyme methylates accessible DNA at GpC sites, whereas nucleosome bound DNA is inaccessible and remains refractory to GpC methylation. PCR amplicons were cloned and individual molecules isolated by colony PCR for sequencing, as described above. Regions of M.*Cvi*PI inaccessibility of ≥150 bp were identified as nucleosome occupied.

## Supporting Information

Figure S1 Epigenetic profiling of the *MLH1* promoter in SW620 cells following decitabine exposure. **A**, qRTPCR results showing *MLH1* gene expression in SW620 cells normalized to *GAPDH*. **B**, Bisulfite pyrosequencing showing average percentage methylation levels across 5 CpG sites located immediately upstream of the *MLH1* annotated TSS. **C**, ChIP qPCR results showing enrichment of H3K4me3 and H3K9ac at the promoter regions of *MLH1* and *GAPDH*. Error bars = SD.
(EPS)

Figure S2 Decitabine exposure leads eviction of nucleosomes from the *MLH1* promoter. Relative nucleosome levels in RKO (A–G) and SW620 (H) cells following decitabine exposure at the indicated time points. Positions of the regions assayed within the *MLH1* promoter are shown in Figure 3A. Error bars = SD.
(EPS)

Table S1 PCR primer sequences for the different assays used in this study. Supplementary references are listed in Supplementary text file S1.
(DOCX)

Text S1 Supplementary references related to primer sequences within supplementary table S1.
(TXT)
