# Supplementary material for: Reassembly of Nucleosomes at the MLH1 Promoter Initiates Resilencing Following Decitabine Exposure
Source: PLoS Genet. 2013 Jul 25;9(7):e1003636. doi: 10.1371/journal.pgen.1003636 (PMC3723495; doi:10.1371/journal.pgen.1003636)
Supplement: Table S1 — PCR primer sequences for the different assays used in this study. Supplementary references are listed in Supplementary text file S1. (DOCX) [file pgen.1003636.s003.docx]

**SUPPLEMENTARY TABLE S1**

| Assay | Gene/Region | Sequence (5’-3’) | DNA Strand | Reference |
| --- | --- | --- | --- | --- |
| qRTPCR | *MLH1* | TAGCCACGAGGAGAAAAGCTTT | Forward | This study |
|  |  | CGACTAACAGCATTTCCAAAGA | Reverse |  |
|  | *GAPDH* | tgttgccatcaatgacccctt | Forward |  |
|  |  | ctccacgacgtactcagcg | Reverse |  |
|  | *SDHA* | TGGGAACAAGAGGGCATCTG | Forward | [28] |
|  |  | CCACCACTGCATCAAATTCATG | Reverse |  |
| Bisulfite sequencing and pyrosequencing | *MLH1* | GGTATTTTTGTTTTTATTGGTT | Forward | [21] |
|  |  | ACTCTATAAATTACTAAATCTCTT | Reverse |  |
|  |  | AAAAAYGAATTAATAGGAA | Sequencing (Pyro) |  |
| MNase digestion and qPCR | Region I | CTACGATGAGGCGGCGAC | Forward | [14] |
|  |  | GACCCAGCGTTATTTGGTGGT | Reverse |  |
|  | Region II | CCTCAGCAGAGGCACACAAG | Forward |  |
|  |  | AATACGAAATATCCAGCCAATAGGA | Reverse |  |
|  | Region III | CAATAGGAAGAGCGGACAGC | Forward | This study |
|  |  | TCTTCGTCCCTCCCTGAAG | Reverse |  |
|  | Region IV | CAGCAACCCACAGAGTTGAGAA | Forward | [14] |
|  |  | GCGGCAGCTATTGATTGGA | Reverse |  |
|  | Region V | CGTAAGCTACAGCTGAAGGAAGAA | Forward |  |
|  |  | CGTCTAGATGCTCAACGGAAGTG | Reverse |  |
|  | Region VI | ACGTTTCCTTGGCTCTTCTG | Forward | This study |
|  |  | TTCACCACTGTCTCGTCCAG | Reverse |  |
|  | Region VII | GGCTGGACGAGACAGTGGTG | Forward | [14] |
|  |  | CAGTTCTCAATCATCTCTTTGATAGCA | Reverse |  |
|  | Region VIII | TGTAAGTGGAGGAATATACGTAGTGTTGT | Forward |  |
|  |  | TCGCATGTTCTGCATACATAATTTT | Reverse |  |
|  | Region IX | CTGTGTTACTGTTTTCTTGCTTTTCAT | Forward |  |
|  |  | GAATTGTGCCTTTGGATGTGAAC | Reverse |  |
| ChIP | *MLH1*  (Region VII) | GGCTGGACGAGACAGTGGTG | Forward |  |
|  |  | CAGTTCTCAATCATCTCTTTGATAGCA | Reverse |  |
|  | *GAPDH* | TACTAGCGGTTTTACGGGCG | Forward | This study |
|  |  | TCGAACAGGAGGAGCAGAGAGCGA | Reverse |  |
|  | *MYOD1* | CCGCCTGAGCAAAGTAAATGA | Forward |  |
|  |  | GGCAACCGCTGGTTTGG | Reverse |  |
| NOMe-Seq | Region N1 | AGAAGYGYTAAGYATTTTTTTYGYTTTGYGYTAGATT | Forward | This study |
|  |  | TCCACTTACACTCCAAACAACCCTTAAAAAATC | Reverse |  |
|  | Region N2 | AGATTATTTTAGYAGAGGYATATAAGYTTGGTTTT | Forward |  |
|  |  | CCAAAAAARCCAAAAAAACATCTAAATRCTCAACA | Reverse |  |
